# Inequalities in children’s exposure to alcohol outlets in Scotland: a GPS study

**DOI:** 10.1186/s12889-022-14151-3

**Published:** 2022-09-15

**Authors:** Fiona M. Caryl, Jamie Pearce, Rich Mitchell, Niamh K. Shortt

**Affiliations:** 1 MRC/CSO Social and Public Health Sciences Unit, School of Health & Wellbeing, University of Glasgow, Glasgow, UK; 2 Centre for Research On Environment, Society and Health, School of GeoSciences, University of Edinburgh, Edinburgh, UK

**Keywords:** Alcohol availability, Socioeconomic status, Activity space, Youth

## Abstract

**Background:**

Alcohol use is a leading cause of harm in young people and increases the risk of alcohol dependence in adulthood. Alcohol use is also a key driver of rising health inequalities. Quantifying inequalities in exposure to alcohol outlets within the activity spaces of pre-adolescent children—a vulnerable, formative development stage—may help understand alcohol use in later life.

**Methods:**

GPS data were collected from a nationally representative sample of 10-and-11-year-old children (*n* = 688, 55% female). The proportion of children, and the proportion of each child’s GPS, exposed to alcohol outlets was compared across area-level income-deprivation quintiles, along with the relative proportion of exposure occurring within 500 m of each child’s home and school.

**Results:**

Off-sales alcohol outlets accounted for 47% of children’s exposure, which was higher than expected given their availability (31% of alcohol outlets). The proportion of children exposed to alcohol outlets did not differ by area deprivation. However, the proportion of time children were exposed showed stark inequalities. Children living in the most deprived areas were almost five times more likely to be exposed to off-sales alcohol outlets than children in the least deprived areas (OR 4.83, 3.04–7.66; *P* < 0.001), and almost three times more likely to be exposed to on-sales alcohol outlets (OR 2.86, 1.11–7.43; *P* = 0.03). Children in deprived areas experienced 31% of their exposure to off-sales outlets within 500 m of their homes compared to 7% for children from less deprived areas. Children from all areas received 22—32% of their exposure within 500 m of schools, but the proportion of this from off-sales outlets increased with area deprivation.

**Conclusions:**

Children have little control over what they are exposed to, so policies that reduce inequities in alcohol availability should be prioritised to ensure that all children have the opportunity to lead healthy lives.

**Supplementary Information:**

The online version contains supplementary material available at 10.1186/s12889-022-14151-3.

## Background

Alcohol use is the leading risk factor for preventable morbidity, disability, and mortality in young people [1], accounting for one in five (19%) deaths in the 15—19 age group in Europe [2]. Alcohol use is also a key driver of rising health inequalities, having a disproportionate impact on people of low socioeconomic status (SES) [3–5]. Despite much of the burden of alcohol-related harm falling on adults, the foundations of damaging health behaviours are often established in childhood. Adolescent alcohol use increases the risk of problem use in adulthood [6–8], so reducing alcohol use during adolescence may help prevent the health consequences of alcohol use and their inequalities.

Age at first use of alcohol—particularly before 15 years—is a powerful predictor of problem alcohol use in adolescence and adulthood [6–8]. In many countries, however, alcohol use starts before the age of 15. In Europe, a third of children (33%) have used alcohol at age 13 or younger [9]. In Scotland—where stark inequalities in alcohol-related morbidity and mortality are growing [10]—a third (36%) of 13-year-olds reported having tried alcohol and half (53%) of those who had ever had alcohol had been drunk at least once [11]. Despite policies to prevent children from accessing alcohol, such as age restrictions on purchases and making it illegal to supply a minor, a significant proportion start experimenting with alcohol at a very young age.

Several factors are associated with alcohol use in young people, including social contexts both inside and outside the home, as well as built environment and media environments [8, 12–15]. Increasing evidence shows that neighbourhood availability of alcohol is associated with alcohol use in adolescence [16–21], including early adolescence (12—14 years) [22–24]. Age-restrictions on alcohol products mean the association between alcohol availability and use is unlikely to be linked to children directly purchasing to alcohol products. Instead, the ubiquitous presence of alcohol outlets—and associated marketing—in children's environments may normalise alcohol as an every-day product, shift social norms in acceptability and use, and shape children’s knowledge, attitudes and beliefs [25–27]. This is supported by longitudinal evidence, which suggests that exposure of children to alcohol marketing—including in-store alcohol displays—influences alcohol use in mid-adolescence and increases risks of early initiation of use [15, 28].

Neighbourhood availability of alcohol is socially patterned, with disproportionately greater densities of alcohol outlets concentrated in areas of socioeconomic deprivation [29–33]. Yet while alcohol-related morbidity and mortality are also higher in disadvantaged socioeconomic groups [30], gradients in alcohol use are small or lacking (known as the ‘alcohol harm paradox’) [4]. An explanation for this is that while alcohol use is associated with harm for all socioeconomic groups, it disproportionately affects those of low SES [5]. Evidence also suggests that vulnerability to alcohol environments is not equal across individual characteristics (e.g., SES, age, sex); alcohol outlet density is strongly associated with harmful alcohol use in low socioeconomic groups, but not in high socioeconomic groups [34]. Hence individuals in low socioeconomic groups are more likely to live in areas of high deprivation with high alcohol availability; are more vulnerable to alcohol availability influencing their use; and face greater risks of alcohol-related harm related to use.

Children form a particularly vulnerable group to alcohol risk environments because it is during this formative stage, in which their brains are still developing, that their attitudes towards, and understanding of, alcohol is shaped [27]. Children have more limited independent mobility than adults—they spend most of their out-of-school time a short distance from home and often only leave the home neighbourhood to go to school [35, 36]—which makes them reliant on their local environment. Children from lower socioeconomic groups are even more constrained by their local environment [37], and more likely to walk to school [38], making them even more vulnerable to the risks presented. Given the potential intersection of vulnerability by age and SES (at individual- and area-levels), there is a surprising lack of studies examining inequalities in exposure to alcohol environments focusing specifically on children [25]. Such data could be used to strengthen demands to protect child environments from ubiquitous alcohol availability.

Reducing alcohol availability is cost-effective strategy for decreasing alcohol use and associated harm [15]. However, empirical evidence to support policy interventions has been limited by inconsistent findings from availability studies, which has been blamed on the measures used to quantify exposure [15, 39, 40]. Alcohol outlet density is often measured at an aggregate level as the number of outlets within a fixed area, such as an administrative boundary [17, 24, 41, 42] or residential buffer [18, 19, 21, 43]. Such measures are susceptible to ecological bias, in which all individuals are attributed the same aggregate level of exposure; the modifiable areal unit problem, in which different aerial boundaries result in different aggregations; and the “local trap”, in which only the local environment, such as the residence, is considered meaningful [44–46]. However, individual spatial routines are highly complex; people move outside of their neighbourhood on a daily basis for work, leisure and other routine activities [47]. Indeed, failure not to recognise the spatial range of individuals’ lives has been identified as a limitation in current alcohol availability research [40].

Recognising that fixed residential measures are not an adequate representation of the environments to which individuals are exposed, exposure research has advanced to measure exposure within an individual-level ‘activity space’ (i.e. the set of places visited through routine activities) [44, 48–50]. Exposure to alcohol environments within individual activity spaces measured using Global Positioning Systems (GPS) data are more strongly associated with behavioural outcomes than those within administrative areas or residential buffers [51, 52]. However, GPS studies are often restricted to small sample sizes, raising concerns about representation [51, 53, 54]. Concerns have also, rightly, been raised about the representation of individuals of low SES in GPS-based exposure studies [55].

Individual-level exposure to alcohol is a product of area-level alcohol availability—which is driven by area deprivation [30]—and individual mobility. In this study, we compare individual exposure to alcohol outlets within the GPS-derived activity spaces of children across a gradient of area deprivation, while controlling for factors affecting mobility. Although our sample, aged 10–11 years old, has not (usually) begun experimenting with alcohol, they represent the age group immediately preceding that in which alcohol initiation often begins. Quantifying exposure at this stage will inform longitudinal research with the same cohort. Crucially, using GPS-based measures we can identify *where* exposure occurs relative to children’s two most visited settings (home and school). This contextualises understanding of exposure, which could be used to inform policy.

## Methods

### Study aims

Our study had three aims:

- i. Determine if the proportion of children exposed to any alcohol outlets varied by area-level socioeconomic deprivation.
- ii. Determine if the proportion of a child’s GPS locations exposed to alcohol outlets varied by area-level socioeconomic deprivation.
- iii. Determine if the relative proportion of a child’s exposure to alcohol outlets that occurred near their home and/or school varied by area-level socioeconomic deprivation.

### Sample

We used secondary data from children in the ‘Studying Physical Activity in Children’s Environments across Scotland’ (SPACES) study [56] who were recruited from the Growing Up in Scotland (GUS) study—a nationally representative longitudinal cohort study originating in 2005. From a possible 2,402 children who participated in GUS 2014/2015 interviews (when the children were aged 10—11 years old), 2,162 (90%) consented to be approached by SPACES researchers, of which 51% (*n* = 1,096) consented to take part in SPACES.

### Location measurement using global positioning system (GPS) device

SPACES participants were provided with an accelerometer (ActiGraph GT3X +) and a waist-mounted GPS device (QstarzSTARZ BT-Q1000XT; Qstarz International, Taiwan) between May 2015 and May 2016, and asked to wear them during waking hours over eight consecutive days. SPACES inclusion criteria required at least four weekdays of accelerometer data and one day of weekend data, resulting in a subset of 774 children. Of these, we used data from children who provided at least one hour of GPS data (> 360 GPS locations) per day.

### Alcohol outlet data

The locations of outlets licensed to sell alcohol (*n* = 16,619) for use on the premises (“on-sales”: *n* = 11,515; 69%) and off the premises (“off-sales”: *n* = 5,104) for 2016 were obtained from local Licensing Boards (*n* = 36) across Scotland. On-sales outlets include businesses such as bars, clubs, restaurants, and cafes. Off-sales outlets include business such as liquor stores, supermarkets, and convenience stores. Locations for each licensed premise were provided as street addresses that we converted to geocoded coordinates (i.e. latitude/longitude) using the ‘ggmap’ R package [57].

### Socioeconomic information

We assigned an area-level measure of deprivation to each child based on their residential datazone (small area census geography containing populations of between 500 and 1,000 residents) using the Income Domain of the 2016 Scottish Index of Multiple Deprivation (SIMD) (Scottish Government 2012). The SIMD is made from seven domains that characterise the social, economic, and physical environment in the area, including aspects such as education and crime. The Income domain was chosen over the overall SIMD because the overall measure includes an element of retail accessibility. The Income domain indicates the proportion of population in each area experiencing income deprivation as measured by receipt of means-tested benefits and government support. Eligibility for means tested benefits is based on income and savings, and benefits are used to top-up income if it is below a certain level. The datazone income ranks were grouped into quintiles (IncQ1 = most deprived, IncQ5 = least deprived). Data on race/ethnicity were not provided, but the GUS cohort, of which this sample were a representative subset, was 96% white.

### Control variables

Individual-level exposure to alcohol is a product of area-level alcohol availability and individual mobility. So in addition to area deprivation, we included several controls that have been shown to influence children’s activity patterns in previous research using SPACES data [58]. Specifically, we classified children by sex; the season in which they were tracked, and whether their residence was in an urban or rural area. We did not include household income as this was not found to influence activity [58]. We classed two seasons corresponding with daylight savings (winter: 25 October 2015—27 March 2016). For rurality we used the Scottish Government’s six-category classification system, which considers both population size of the settlement and remoteness/accessibility (based on drive time to the nearest settlement with a population of 10,000 people or more) [59]. To ensure sufficient sample sizes within groups, we dichotomised the six-category classification system into two categories (urban, rural), each comprising three of the original classes.

### Data linkage

GPS devices recorded child locations at 10-s intervals. Longitude and latitude from GPS locations and outlet locations were projected to the British National Grid coordinate reference system (CRS) (epsg: 27,700) to correspond with other spatial data (i.e., SIMD and urban–rural classifications). The Euclidean distance from every GPS location (*n* = 15.9 M) to every alcohol outlet location was measured using the ‘sf’ R package [60] to determine the nearest outlet to each GPS location. The Euclidean distance from each GPS location to each child’s home and their school location was also measured. We identified whether nearest outlet held an on- or off-sales licence and classed GPS locations as ‘exposed’ when the distance to the nearest alcohol outlet was ≤ 10 m. The 10 m threshold was used to reflect the accuracy of GPS receivers, which varies by mode of travel (walking, bicycle, vehicle) and environment (number and height of adjacent buildings). For example, walking in urban canyons has lower accuracy (mean 11.5 m, SD 14.0 m) compared to walking in open areas (mean 5.1, SD 10.2 m); however, 78.7% of GPS locations fall within 10 m of expected location across travel modes and environments [61].

### Outcomes

#### Proportion of children exposed

We created a binary variable indicating if each child had been exposed to any alcohol outlet, from which we could calculated the *proportion of children exposed*.

#### Proportion of GPS exposed

For each child, we quantified the *proportion of GPS exposed* to either an on- or off-sales alcohol outlet. To do this, we used a count of GPS locations exposed to 1. on-sales outlets; and 2. off-sales outlets, as a proportion of total count of GPS locations (e.g., number of GPS exposed to alcohol outlets / total GPS number).

#### Relative exposure within home and school settings

For each child, we quantified the *relative proportion of exposure* occurring within their home or school settings. To do this, we used a count of GPS exposed to on-sales outlets within distance 300 m, 400 m and 500 m bands of home by the total count of GPS exposed to alcohol outlet (i.e., number GPS exposed to on-sales within home setting / number of GPS exposed). We repeated this with GPS exposed to on-sales outlets within school setting. We then repeated both home and school measures on GPS exposed to off-sales outlets resulting in four outcomes; relative proportion of exposure to: 1. On-sales within home settings; 2. Off-sales within home settings; 3. On-sales within school settings; 4. Off-sales within school settings.

The distance bands chosen to delineate settings have been used in other studies quantifying exposure around residential and school locations of children [25, 62–64]. We quantified the distribution of time spent (i.e., proportion of GPS) within each distance band exclusive to home and school and conducted a sensitivity analysis on the effect of distance band choice. However, as it was possible for a GPS location to fall within distance of both home and school (e.g., a GPS could within 500 m of home and school) we classed GPS occurring within both settings separate from those occurring exclusively within one setting when quantifying relative exposure within settings.

For analysis of both settings, we only included data for children who had been exposed (*n* = 659). For the home setting analysis, we removed data from four children whose residential location co-occurred with an alcohol outlet location (e.g., child lived above a shop) (*n* = 655). For the school setting analysis, we removed data from ten children who were never located within 500 m of school (*n* = 649). SPACES sampling aimed to avoid school breaks, but children who were never located on school premises were assumed to have been participating in the study outside of normal school attendance. The distribution of the sample by area deprivation in each subset did not differ from the full dataset.

### Data analysis

#### Descriptive statistics

Descriptive statistics were given for covariates (area deprivation, urban/rural classification, season, sex) along with the number of GPS included in the analyses. Sample weights were applied to all descriptive and statistical analysis. Sampling weights were applied to allow for non-consent to contact, non-consent, and non-compliance of those invited to take part. We used weighted means (from the ‘survey’ R package [65, 66]) to find the average proportion of exposures to on- and off-sales outlets within 500 m of home or school settings by area deprivation.

#### Statistical analysis

Each dependent variable (i.e., 1. proportion of children exposed to alcohol outlet; 2. proportion of GPS exposed to on-sales; 3. proportion of GPS exposed to off-sales) was fitted with a generalised linear model (GLM) using the ‘survey’ R package with a quasibinomial distribution to account for counts (i.e., number of exposed GPS) becoming non-integer after weighting. Fixed effects included area deprivation quintile (as factor), and binary measures of urbanicity, sex, and season. Sampling weights and strata were applied to all models to account non-consent and non-compliance of those invited to take part along with the clustered and stratified nature of the sampling design [65].

Fully adjusted logistic regression results were output as Odds Ratios to interpret difference in odds by area deprivation quintile (using the least deprived quintile as the reference level). Models compared the *observed proportion* of GPS exposed. To interpret what model coefficients meant in real-world terms we extracted coefficients (i.e., log-odds) and back transformed them to the response scale (i.e., probability of GPS exposed; which is essentially the *expected proportion* of GPS exposed). Predicted probability (i.e., expected proportion) of GPS exposed was then used to predict mean duration exposed in a week of GPS wear.

## Results

A total of 688 children were included in the analysis (Table 1). Of children included in the study, 96% had 4 or more days with GPS, and 86% had 7 days (Supplementary Fig. 1). The median total number of GPS locations per child was 24,280 (IQR range 7634), equivalent to 67 (IQR 55—76) hours of wear. Similar numbers of GPS were collected across sample covariates (Table 1).

**Table 1:** Sample distribution across covariates (weighted) and sampling effort of *n* = 688 participants

Covariate	%	Median (IQR) GPS locations per child
Income deprivation (area-level)		
Most Deprived	22.9	22,553 (17,975–25,680)
IncQ2	16.5	23,775 (18,341–27,277)
IncQ3	17.9	24,637 (19,625–28,042)
IncQ4	19.4	24,358 (20,739–27,522)
Least Deprived	23.3	24,395 (20,727–27,038)
Sex		
Male	45	24,259 (20,169–27,380)
Female	55	24,304 (19,595–27,429)
Urban/Rural Class		
Urban	80.3	24,067 (19,577–27,021)
Rural	19.7	25,103 (21,638–28,116)
Season		
Summer	49.4	21,324 (24,918–27,900)
Winter	50.6	18,957 (23,027–26,690)
Total	100	24,281 (19,757–27,392)

### Inequalities in exposure

In total, 591 (86%) of children were exposed to alcohol outlets during the study, however, the proportion of children exposed was not found to differ by area-level deprivation (Table 2, Model 1).

**Table 2:** Odds ratios (95% CI) from quasibinomial generalized linear models. Model 1 compares proportion of children who were exposed to any alcohol outlet by area-level deprivation. Model 2 compares observed proportion of GPS locations from each child exposed to off-sales and on-sales alcohol outlets by area-level deprivation. (IncQ1 = most deprived)

	Model 1	Model 2	
		Off-sales	On-sales
Least deprived (IncQ5)	Ref	Ref	Ref
IncQ4	0.91 (0.36–2.27)	1.36 (0.87–2.11)	1.68 (1.05–2.69) *
IncQ3	1.20 (0.29–4.90)	2.15 (0.83–5.58)	2.16 (1.08–4.27) *
IncQ2	0.84 (0.12–6.06)	3.17 (2.29–4.39) ***	3.09 (1.86–5.15) ***
Most deprived (IncQ1)	1.26 (0.33–4.89)	4.83 (3.04–7.66) ***	2.86 (1.11–7.43) *
Urbanicity (urban)	Ref	Ref	Ref
Urbanicity (rural)	0.61 (0.23–1.77)	0.66 (0.38–1.16)	0.97 (0.51–1.84)
Season (winter)	Ref	Ref	Ref
Season (summer)	0.64 (0.23–1.77)	1.79 (1.18–2.71) **	1.26 (0.68–2.30)
Sex (male)	Ref	Ref	Ref
Sex (female)	0.84 (0.33–2.14)	1.37 (0.88–2.14)	1.36 (0.72–2.55)
N	688	688	688
Pseudo R2	0.02	0.24	0.07

Pseudo R2 = 1 – (Residual Deviance / Null Deviance)

^***^ *p* < 0.001; ** *p* < 0.01; * *p* < 0.05

The predicted probability that a GPS location was within 10 m of *any* type of alcohol outlet (i.e., exposed) was 0.0079 (95% CI 0.0045—0.0113). Assuming the GPS is representative of where children spend their time, this means that 0.08% of children’s time was exposed to alcohol outlets. In a 67-h period (i.e., median GPs wear time across all children) this equated to 28.4 (23.4—33.5) minutes of exposure (i.e., 4020 min * 0.0079). Approximately half (47%) of this likelihood (0.0037, 0.0021—0.0053) was from off-sales alcohol outlets, which is higher than expected given their lower availability (i.e., 31% of all outlets held off-sales licences).

**Fig. 1:**
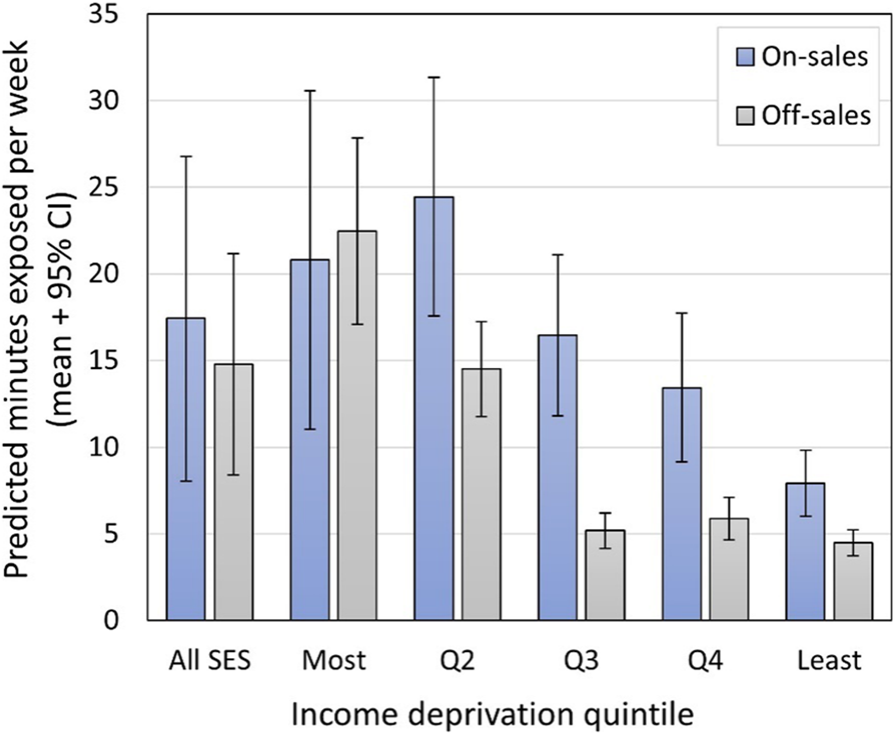
Duration (minutes) of exposure for children by area-level income-deprivation (mean ± 95% CI). Exposure duration predicted for 67-h period (based on the median number of GPS collected per child) after adjusting for control variables

Comparison with ORs indicated that there were inequalities in the probability of exposure to off-sales and on-sales alcohol outlets (Table 2, Model 2). Specifically, the probability of being exposed to off-sales alcohol outlets was 4.83 (3.04–7.66) and 3.17 (2.29–4.39) times greater for children living in the two most deprived areas (IncQ1 and IncQ2) than children in the least deprived areas (IncQ5: Table 2). This means that in a 67-h period we would expect children in the most deprived areas to be exposed to off-sales alcohol outlets for 22.5 (17.1—27.8) minutes compared to 4.5 (3.7—5.2) for children in the least deprived areas (Fig. 1). The probability of children from IncQ 1—4 being exposed to on-sales alcohol outlets were all higher than those in the least deprived areas (IncQ5: Table 2). However, it was children in the second most deprived areas (IncQ2) who had the highest probability of being exposed to on-sales outlets (equivalent to 24.4, 17.6—31.3 min: Fig. 1).

### Relative exposure within home and school settings

The relative proportion of exposure within home and school settings showed similar patterns across 300 m, 400 m, and 500 m distance bands (Supplementary Table 1). We present results using the 500 m distance band here because this accounted for a greater proportion of their time. The mean proportion of time spent within 500 m of home was 56% (55—57%) across individuals by area deprivation, with 53% (51—54%) of tine spent within 500 m of school. Note that settings were not mutually exclusive when determining time spent there, so GPS could be counted in both settings. There was little variation in mean proportion of time spent within 500 m of schools by area deprivation (most deprived:55%, 51—59%; least deprived: 51%, 48—53%), but children in the most deprived areas spent slightly more time near home (61%, 58—65%) than those from the least deprived areas (54%, 52—56%).

**Fig. 2:**
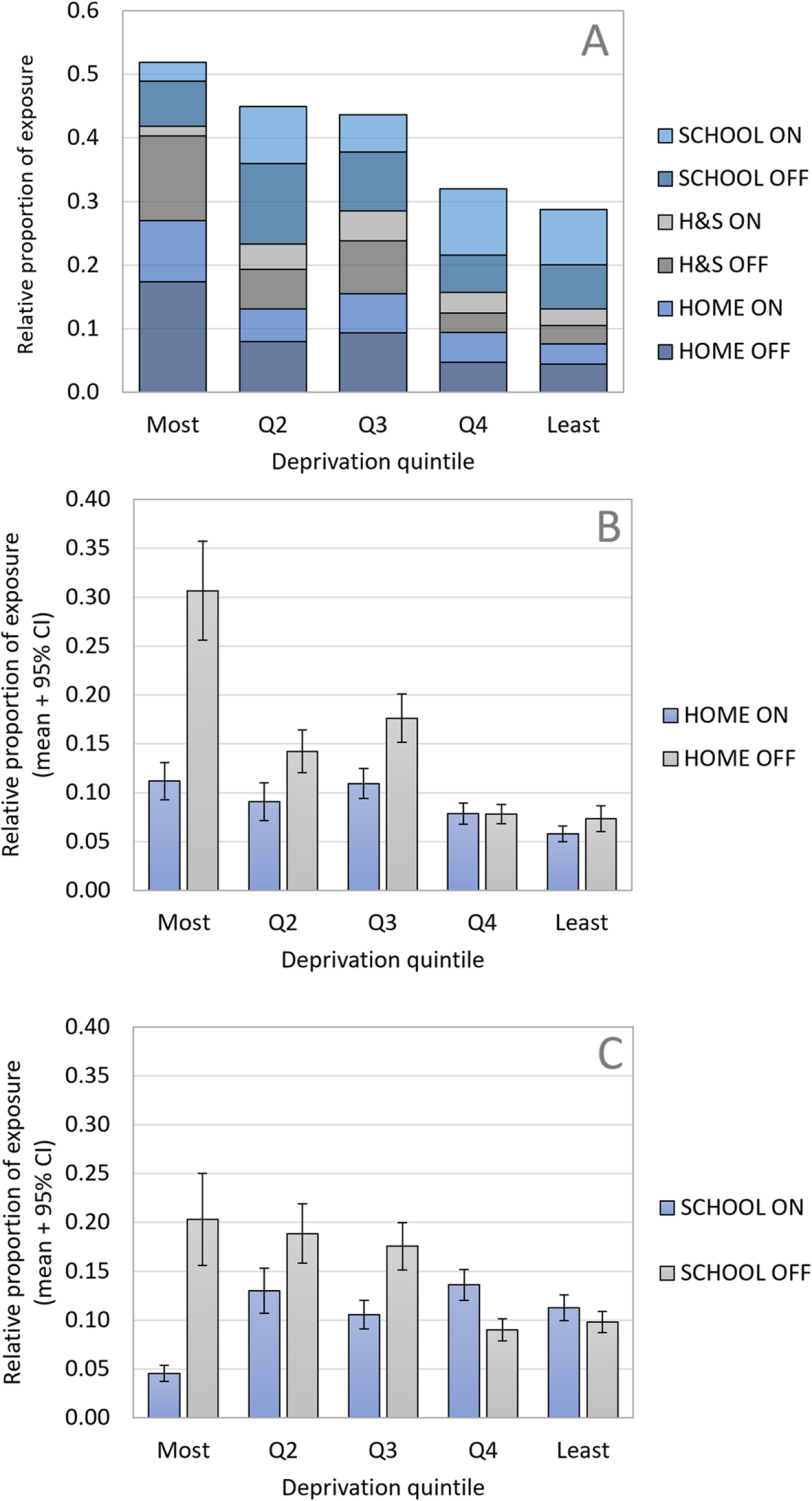
Mean proportion of exposure to alcohol outlets occurring within home and school settings. **A** Disaggregated GPS exposures overlapping between both settings (i.e., 500 m of home and school) are categorised as HS; (**B**) Aggregated GPS exposures within home setting (i.e., home + HS); (**C**) Aggregated GPS exposures within school setting (i.e., school + HS)

We disaggregated GPS that fell exclusively within 500 m of home *or* school from those falling within 500 m of both home *and* school (Fig. 2A). This indicated there was a gradient in the proportion of GPS falling within both settings, which declined as area deprivation lessened (i.e., children in deprived areas had more exposed GPS co-occurring within 500 m of home *and* school). Children in the most deprived areas experienced half (51.9%) of all their exposure within 500 m of home and/or school, most of which (72.7%) was from off-sales outlets (Fig. 2A). By contrast, children in the least deprived areas experienced less than a third (28.7%) of their exposure within 500 m of home and/or school, half of which (49.7%) was from off-sales outlets (Fig. 2A). For ease of communication, we henceforth report results aggregated by setting (e.g., home setting reported as results exclusive to home setting plus those exclusive to home *and* school: Fig. 2B and C).

Relative exposure to on- and off-sales outlets within home settings (Fig. 2B) was highest for children in the most deprived areas (41.9%) and lowest in the least deprived areas (13.1%). Almost a third (30.7%) of all exposure experienced by children in the most deprived areas came from off-sales outlets within 500 m of home. By contrast, off-sales outlets within 500 m of home accounted for just 7.3% of the total exposure for children in the least deprived areas. Across deprivation quintiles, 21.1—31.9% of relative exposure occurred within school settings (Fig. 2C). However, this was predominantly from off-sales outlets for children in the three most deprived quintiles (most deprived = 81.7%; IncQ2 = 59.2%; IncQ3 = 62.4%). Children in the least deprived quintile were equally exposed to on- and off-sales outlets within school settings (53.5% on-sales), whereas those in IncQ4 got most (60.2%) of their exposure within school settings from on-sales outlets.

## Discussion

Scotland has marked social gradients in alcohol-related hospitalisations, morbidity, and mortality that contribute to widening socioeconomic health inequalities [10, 30]. Reducing alcohol availability has been highlighted as a cost-effective strategy to reduce alcohol use and harm [15, 26]. Given the strong link between use of alcohol in childhood and alcohol-related harms in adulthood [6, 7, 67], along with the differential impact that alcohol availability has on different socioeconomic groups [34], our findings could identify policy levers to decrease inequalities in alcohol exposure and, ultimately, harm. Crucially, our sample (*n* = 688) represented children across a socioeconomic gradient, at a vulnerable age—just prior to first experimenting with alcohol, which in Scotland is 13 years old [11]. As such, this study represents an advance in our understanding of how alcohol risk environments vary at the intersection of two vulnerable (yet understudied) characteristics [27, 34]. We found that the proportion of children exposed to alcohol outlets did not differ by area deprivation. However, the proportion of time children were exposed to alcohol outlets showed stark inequalities. Children living in the most deprived areas were five times more likely to be exposed to off-sales outlets than children from the least deprived areas. These children were also three times more likely to be exposed to on-sales outlets, although the relationship was not linear—children in the second most deprived areas had the highest probability of exposure. Children in the most deprived areas received half (52%) of their total exposure within 500 m of their homes and schools, predominantly from off-sales outlets (73%). By contrast, home and school settings accounted for less than a third (29%) of children’s exposure in the least deprived areas, which was equally from on- and off-sales outlets. Indeed, almost a third (31%) of all exposure experienced by children in deprived areas was attributable to off-sales outlets within 500 m of their homes, compared to just 7% for the least deprived areas.

On- and off-sales alcohol outlet densities have different socioeconomic drivers [29], which explains some of the patterns we observed by area deprivation. For instance, off-sales alcohol outlets tend to proliferate in areas of high deprivation; whereas on-sales outlets proliferate in areas of medium deprivation; and areas of low deprivation have the lowest numbers of both outlet types [29]. This is supports our finding that children in IncQ1 had the greatest exposure to off-sales outlets, while those in IncQ2 had the greatest exposure to on-sales outlets; and those in IncQ5 had the least exposure to either outlet type. However, the inequalities in exposure to off-sales outlets we found were far larger than those previously reported for Scotland [29]. Comparing densities of outlet type within census tracts, Shortt et al. found off-sales densities were twice as high in the most deprived areas than the least [29] whereas we found a fivefold difference. This is supported by previous research that found low correlation between exposure to alcohol environments measured within individual activity spaces versus administrative boundaries [52, 54]. Children spend most of their time a short distance from home and leave their home neighbourhoods primarily to attend school [35, 36]. Our data suggest that children in deprived areas spent slightly more time within 500 m of home (61%, 58—65%) than those from the least deprived areas (54%, 52—56%). While previous research shows children living in areas of higher deprivation are also more likely to walk than children living in areas in areas of lower deprivation [38]. It is therefore not surprising that inequalities in alcohol outlet density are amplified once individual mobility is accounted for.

We found that exposure risk within school settings was also socially patterned. Children in the three most deprived quintiles received relatively more exposure to off-sales outlets within school settings than those in less deprived areas. Secondary (high) schools in deprived areas have higher densities of alcohol outlets around them than schools in less deprived areas, prompting calls to limit alcohol availability around schools [64]. We are unaware of studies reporting densities of alcohol outlets around primary (elementary) schools. However, we found that children from more deprived areas are more likely to attend schools that are closer to their homes than children from less deprived areas. Children in the most deprived areas experienced an average 13% of their exposure within 500 m of home *and* school compared to 2% for children in the least deprived areas. Hence policy interventions to reduce alcohol availability around primary (elementary) schools might be effective at reducing availability around the homes of children in deprived areas who live close to their schools.

Several studies have found an association between alcohol availability and use in children [12, 22, 39]. Notably, this association was stronger for off-sales alcohol outlets [17, 19, 21] than for on-sales alcohol outlets [19, 24]. Availability of off-sales outlets is positively associated with children’s (age 11–13) exposure to alcohol marketing [25], which influences alcohol consumption in mid-adolescence [28], and increases risks of early initiation of drinking [15]. Our finding that children from deprived areas were most exposed to off-sales is therefore highly problematic. Children are often able to enter off-sales outlets, such as a grocery stores selling alcohol, unaccompanied by an adult, whereas laws prohibit entry of children to many on-sales outlets, such as public houses, without an accompanying adult. Additionally, alcohol products in off-sales outlets, such as grocery stores and supermarkets, are often co-located with products directly accessed by children (e.g., soft drinks and snacks) [68, 69]. So, while we measured proximity of children to alcohol outlets, and not whether they entered those outlets, exposure to off-sales outlets in-and-of-itself comes with implicit additional risks because children are not restricted on entering them and may, in fact, deliberately enter them.

### Research implications

Children have no authority over what they are exposed to, so public policies are needed to address inequalities in the availability of alcohol, particularly off-sales outlets in which alcohol products and marketing are visible in shops visited by children daily. Interventions to reduce children’s exposure to alcohol could include removing—or limiting the number of—licenses to sell alcohol from off-sales outlets visited regularly by children, such as supermarkets, grocery stores and newsagents. These types of outlets tend to proliferate in areas of high deprivation and could therefore be a useful lever for reducing inequalities in exposure [70]. Limiting the number of off-sales licenses granted to premises close to primary (elementary) schools could be a more palatable policy to reduce inequalities [70] with the additional benefit of protecting children’s homes that are near schools. Other interventions could involve reducing visibility of alcohol products within shops visited by children with display bans or segregated areas [69]. In considering options, policymakers must be mindful of policy equity-impacts and determine whether to implement policies targeted at protecting children who are at higher risk versus all children [70].

### Limitations

We classed exposure based on proximity of GPS to retailers using GPS collected at 10-s intervals. It is likely, therefore, that there were instances when a child was within 10 m of an outlet but no GPS location was recorded. However, if outlets were passed frequently (such as walking the same route to school) these outlets should be detected and the rates of undetected outlets should be equally distributed across children. Our methods mean exposures are more likely to be detected when a child has paused or is moving slowly than when they are moving within a vehicle. Exposure is therefore representative of relative time spent exposed given a child’s activity level or mode of transport. Our ability to measure if children entered outlets (as opposed to being within 10 m of them) was prevented by the fact that GPS do not work indoors. We were unable to disaggregate retail types into more granular categories (e.g. supermarkets, pubs, grocery stores), which would improve understanding of the most problematic types out outlets [40]. We did not have access to data on health behaviours or outcomes. However, our sample forms part of a longitudinal study in which alcohol use will be included in future surveys so we will be able to explore how exposure to alcohol in childhood associates with health in adolescence when data become available.

## Conclusions

Children living the most deprived areas—who are most at risk from the harms of alcohol and most vulnerable to local alcohol outlet densities—experience the most exposure to alcohol outlets. Inequalities are particularly attributable to off-sale outlets within 500 m of their homes, and (to a lesser extent), their schools. Policymakers need to urgently address inequalities in alcohol availability if they wish to provide all children with the opportunity to remain alcohol free as they move into adolescence and reduce health inequalities in later life.

## Supplementary Information

**Additional file 1: Supplementary Figure 1. **Proportion of sample returning 4+ days and 6+ days of GPS data, and median number of GPS per individual used in this study. **Supplementary Table 1.** Sensitivity analysis showing how use different distance bands (300m, 400m, 500m) to define home and school settings impacts the relative proportion of exposure attributed to those settings. “H&S” indicates GPS the fell within distance of both home and school. “HOME” and “SCHOOL” categories are exclusive from “H&S”. The socioeconomic distribution for home and school subsets is also shown. **Supplementary Figure 2.** Mean proportion of GPS (95% CI) by distance from home and school (data labels indicate values for all income deprivation quintiles combined).

## Data Availability

The datasets analysed during the current study are not publicly available and restrictions apply to their availability. For further information, please refer to the SPACES study data sharing portal at http://spaces.sphsu.mrc.ac.uk
