# Supplementary material for: Inequalities in children’s exposure to alcohol outlets in Scotland: a GPS study
Source: BMC Public Health. 2022 Sep 15;22:1749. doi: 10.1186/s12889-022-14151-3 (PMC9479265; doi:10.1186/s12889-022-14151-3)
Supplement: Supplementary file 1 — Additional file 1: Supplementary Figure 1. Proportion of sample returning 4+ days and 6+ days of GPS data, and median number of GPS per individual used in this study. Supplementary Table 1. Sensitivity analysis showing how use different distance bands (300m, 400m, 500m) to define home and school settings impacts the relative proportion of exposure attributed to those settings. “H&S” indicates GPS the fell within distance of both home and school. “HOME” and “SCHOOL” categories are exclusive from “H&S”. The socioeconomic distribution for home and school subsets is also shown. Supplementary Figure 2. Mean proportion of GPS (95% CI) by distance from home and school (data labels indicate values for all income deprivation quintiles combined). [file 12889_2022_14151_MOESM1_ESM.docx]

**Supplementary Figure 1: Proportion of sample returning 4+ days and 6+ days of GPS data, and median number of GPS per individual used in this study.**

**
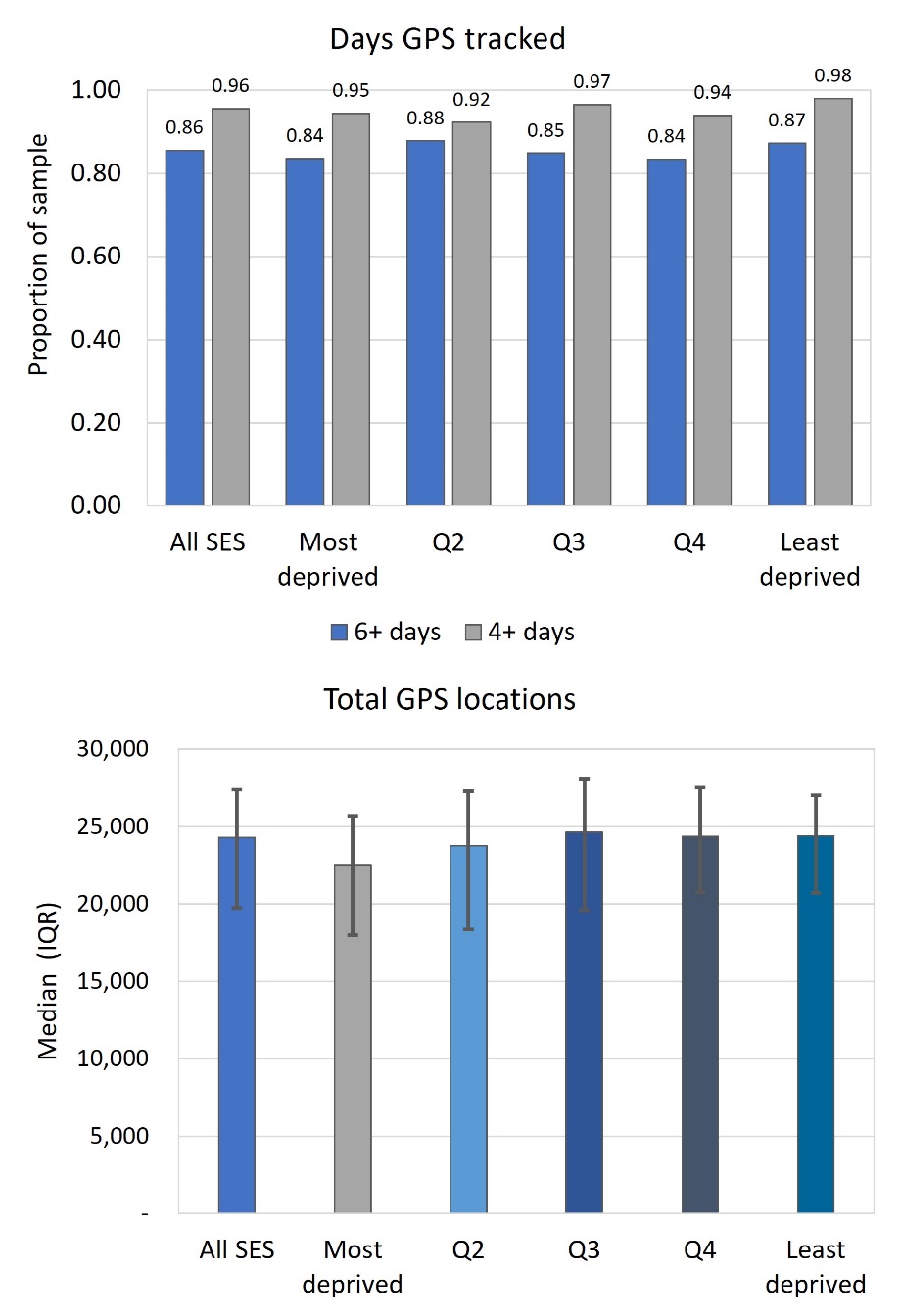
**

**Supplementary Table 1: Sensitivity analysis showing how use different distance bands (300m, 400m, 500m) to define home and school settings impacts the relative proportion of exposure attributed to those settings. “H&S” indicates GPS the fell within distance of both home and school. “HOME” and “SCHOOL” categories are exclusive from “H&S”. The socioeconomic distribution for home and school subsets is also shown.**

| **500m** | **Most deprived** | **Q2** | **Q3** | **Q4** | **Least deprived** |
| --- | --- | --- | --- | --- | --- |
| HOME ON | 0.096 | 0.051 | 0.062 | 0.047 | 0.032 |
| H&S ON | 0.016 | 0.040 | 0.047 | 0.032 | 0.026 |
| SCHOOL ON | 0.030 | 0.090 | 0.058 | 0.104 | 0.087 |
| HOME OFF | 0.174 | 0.080 | 0.093 | 0.047 | 0.044 |
| H&S OFF | 0.133 | 0.062 | 0.083 | 0.031 | 0.029 |
| SCHOOL OFF | 0.070 | 0.126 | 0.093 | 0.059 | 0.069 |
| **400m** |  |  |  |  |  |
| HOME ON | 0.080 | 0.040 | 0.037 | 0.041 | 0.023 |
| H&S ON | 0.007 | 0.033 | 0.045 | 0.031 | 0.022 |
| SCHOOL ON | 0.022 | 0.072 | 0.044 | 0.096 | 0.076 |
| HOME OFF | 0.109 | 0.061 | 0.063 | 0.035 | 0.036 |
| H&S OFF | 0.103 | 0.060 | 0.058 | 0.029 | 0.025 |
| SCHOOL OFF | 0.021 | 0.109 | 0.049 | 0.050 | 0.058 |
| **300m** |  |  |  |  |  |
| HOME ON | 0.060 | 0.031 | 0.044 | 0.035 | 0.026 |
| H&S ON | 0.000 | 0.020 | 0.018 | 0.016 | 0.013 |
| SCHOOL ON | 0.013 | 0.032 | 0.029 | 0.083 | 0.053 |
| HOME OFF | 0.103 | 0.053 | 0.060 | 0.029 | 0.017 |
| H&S OFF | 0.070 | 0.052 | 0.042 | 0.006 | 0.017 |
| SCHOOL OFF | 0.015 | 0.098 | 0.045 | 0.048 | 0.053 |
| Full sample (n=688) | 23% | 17% | 18% | 19% | 23% |
| Home setting (n=655) | 23% | 16% | 18% | 19% | 23% |
| School setting (n=649) | 23% | 17% | 18% | 19% | 23% |

**Supplementary Figure 2: Mean proportion of GPS (95% CI) by distance from home and school (data labels indicate values for all income deprivation quintiles combined).**

**
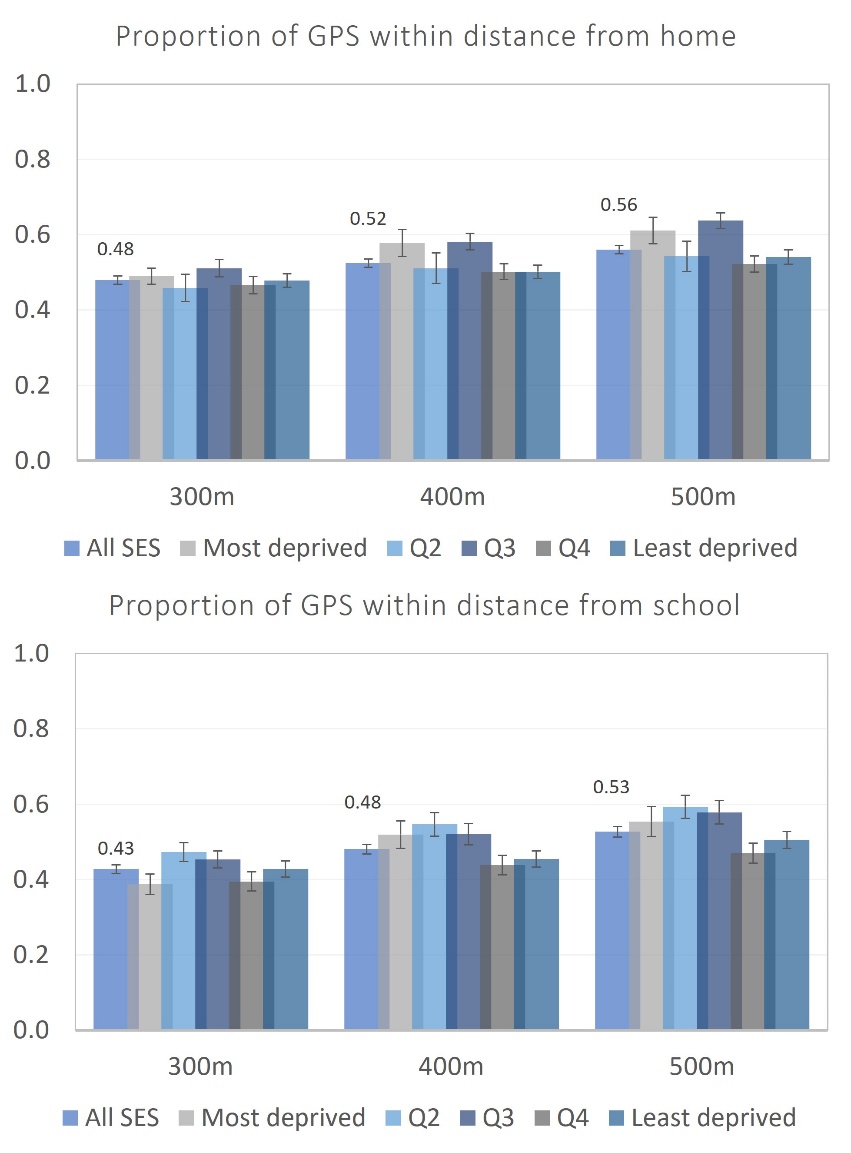
**
